# Determinants of exclusive breastfeeding in rural South India

**DOI:** 10.1186/s13006-018-0178-5

**Published:** 2018-08-29

**Authors:** Holly Nishimura, Karl Krupp, Savitha Gowda, Vijaya Srinivas, Anjali Arun, Purnima Madhivanan

**Affiliations:** 1 School of Public Health, University of California at Berkeley, 50 University Hall Berkeley, Berkeley, CA 94720-7360 USA; 2 Robert Stempel College of Public Health and Social Work, Florida International University, 11200 SW 8 Street, HLS 390W2, Miami, FL 33199 USA; 3 Public Health Research Institute of India, 89/B, Ambika, 2nd Main, 2nd Cross, Yadavagiri, Mysore, 570020 India

**Keywords:** Exclusive breastfeeding, Complementary food, Infant feeding, India

## Abstract

**Background:**

While breastfeeding rates have improved globally, disparities in breastfeeding practices persist particularly in rural and low resource settings. In India, only 56% of Indian mothers practice exclusive breastfeeding (EBF) for the recommended six months. As India leads the world in the number of preterm births, under 5 years of age malnutrition and neonatal mortality, understanding the factors associated with EBF can help improve the nutritional status for millions of infants. We assessed the factors associated with EBF in rural Mysore, India.

**Methods:**

This cross-sectional analysis was nested within a cohort study assessing the feasibility and uptake of mobile prenatal care and HIV counseling and testing intervention in Mysore District. Multivariable logistic regression was used to identify the factors associated with EBF for infants between birth and six months. Exclusive breastfeeding was defined as breastfeeding with no other liquids or breastfeeding substitutes given to infants exceptfor medicine or oral rehydration solution, between birth and 6 months and was assessed at six months postpartum.

**Results:**

We surveyed mothers who delivered in rural Mysore *taluk* between 2008 and March 2011. A total of 1292 mothers participated in the study. The overall breastfeeding rate at six months postpartum was 74.9% and the EBF rate was 48.5%. Factors associated with EBF included higher maternal age (Adjusted Odds Ratio[aOR] 1.04; 95% Confidence Interval [CI] 1.00, 1.09), lower maternal education (aOR1.56, 95% CI 1.10, 2.21), and 7–10 antenatal visits (aOR 1.57; 95% CI 1.09, 2.27). The most common reason for non-exclusive breastfeeding was the mother’s feeling that she did not have enough milk (23.7%). Infants that were not exclusively breastfed were most commonly fed formula/animal milk (42.6%) or castor oil/ghee (18.4%).

**Conclusions:**

Less than half of the mothers in our sample reported exclusive breastfeeding in a rural region of Karnataka, India in the first six months, a rate lower than national and state level rates. Future interventions should evaluate whether antenatal education can improve breastfeeding outcomes. The only modifiable factor was number of antenatal visits. Breastfeeding education should be emphasized at every antenatal visit so that even mothers with fewer than 7–10 antenatal visits can learn the best techniques and benefits of breastfeeding.

## Background

For optimal growth and development, the World Health Organization (WHO) recommends exclusive breastfeeding (EBF) for the first 6 months of life. During this period, no other liquids or breastfeeding substitutes should be given to infants except for medicine or oral rehydration solution [1]. There is evidence to suggest that infants who are breastfed exclusively have 13% reduced risk of mortality compared to non-exclusively breastfed infants in low and middle income countries (LMICs) [2].

While breastfeeding rates have improved globally, disparities in breastfeeding practices persist particularly in rural and low resource settings [3–8]. In LMICs, only 37% of children are breastfed exclusively for the first 6 months of life [9] and India is no exception. According to the National Family and Health Survey-4, on average only 56% of Indian mothers practiced EBF for the full 6 months [10]. As India leads the world in the number of preterm births, under 5 years malnutrition, and neonatal mortality, understanding the factors associated with exclusive breastfeeding can help improve the nutritional status for millions of infants. Furthermore, understanding factors that influence EBF practices can contribute to achieving the United Nations Sustainable Development Goal 3 (SGD3) of reducing neonatal mortality to at least as low as 12 neonatal deaths per 1000 live births by 2030 [11].

To identify factors associated with EBF in rural India, we assessed sociodemographic, birth characteristics, and breastfeeding practices among a cohort of 1292 rural mother-infant dyads in Mysore District in the south Indian state of Karnataka.

## Methods

### Study setting and data collection

The study was conducted between 2008 to 2011 in Mysore District, Karnataka, India. The district has a population of 2,994,744 persons, of which 1,483,538 (49.5%) are female. More than half of Mysore’s residents (58.6%) reside in the district’s 1332 rural villages. Annual per capita income for rural residents is estimated at INR 16,086 [USD $322] and literacy at 63.3%, compared with an all India annual per capita income of INR 38,005 [USD $760] and literacy rate of 74.0%. [12–14] The majority (86%) of residents identify as Hindu and the remaining 14% identify as Muslim or other religions.

This cross-sectional analysis was nested within a cohort study conducted by the Public Health Research Institute of India (PHRII). Methods for the *Kisalaya* cohort study are detailed in Kojima et al. [15]. PHRII staff interviewed expectant mothers before birth, within 15 days after birth, and again at 6 months after birth. At the 6 month follow up period, 1292 women who delivered live infants and consented to be interviewed were administered a pretested standardized questionnaire in *Kannada* language based on National Family Health Survey (NFHS-III) to collect information on a child’s birth history, general health, immunization schedule, breastfeeding status and a woman’s general health, sexual health, birth preparedness following current pregnancy and other sociodemographic characteristics.

### Study measures

We defined exclusive breastfeeding practice as a mother who breastfed her child exclusively for 6 months without supplementation based on the WHO Infant and Young Child Feeding (IYCF) definition. To assess whether mothers were practicing EBF they were asked, “Are you feeding your baby any items other than breast milk?” and “When did you stop breastfeeding your baby exclusively?” in the first follow up survey (15 days after birth) and the second follow up survey (6 month follow up survey). In order to assess factors associated with exclusive breastfeeding, we devised a 13-item questionnaire based on the existing literature. Items assessed sociodemographic characteristics of the mother including age at delivery (years), education (none, primary or > primary), employment (yes or no), gravidity, monthly household income (< 4000, 4001–10,000 or > 10,000 Indian rupees) and religion (Hindu or Muslim/others). We assessed characteristics of the birth including birthweight of infant (gms), antenatal visits (number of antenatal visits), birth attendant (traditional birth attendant, relative, auxiliary nurse midwife, or doctor), and mode of delivery (vaginal or caesarian), infant characteristics including infant gender (male or female), place of delivery (home, government health centers, or private maternity nursing homes), assistance during delivery (yes or no), and satisfaction with infant gender (yes or no). We also assessed reasons for discontinuing EBF, and what mothers used to supplement breastmilk when they discontinued exclusive breastfeeding.

### Statistical analysis

Descriptive statistics were performed using frequencies and proportions for categorical variables. Bivariate analyses were conducted to determine differences in maternal, infant and socio-demographics between mothers who mothers reported exclusive breastfeeding practice versus those who did not using univariate logistic regressions. Multivariable logistic regression analyses were conducted to delineate the factors associated with EBF practices. Regression diagnostics to detect multicollinearity or redundancy were conducted and did not detect any important correlations that warrant omission of variables from the models. We did not include occupation and religion of a mother in the regression model due to absence of variability. Odds ratio and associated 95% confidence interval were calculated to measure associations. All analyses were conducted using STATA version 14.

## Results

### Demographic characteristics

A total of 1292 mother-infant dyads were included in the study. Demographic characteristics of mothers and infants in the cohort are presented in Table 1 and by practice of EBF in Table 2. The mean age of mothers in the study was 20.8 ± 2.75 years. The majority of participants (47.5%) had more than primary school education and were primigravida (53.8%). The primary religion reported was Hindu (99.1%). A little over half (51.3%) of the households earned less than 4000 INR monthly and almost all participants (97.5%) reported being housewives or not employed. The most common mode of delivery was vaginal (83.1%) and more than half (56.7%) of women delivered in a sub-center, primary healthcare facility, or district health center in Mysore district. Births were most commonly assisted by a doctor or nurse (86.2%). While 69.7% of the women received between seven and 10 antenatal visits, 66.3% received breastfeeding assistance. A considerable proportion of births were preterm (42.9%) and 38.6% of infants were low birthweight (< 2500 g). None of the infants in the study were very low birthweight (< 1500 g).

**Table 1:** Sociodemographic characteristics of mother-infant dyads (*n* = 1292)

	Total (*n* = 1292)	
Characteristics	*n*	%
Maternal age, mean (SD)	20.8(2.75)	–
Maternal education		
No education	168	13.0
Primary (1–8 yrs)	511	39.6
More than primary (> 8 yrs)	613	47.5
Religion		
Hindu	1280	99.1
Muslim/others	12	0.9
Employed		
No	1259	97.5
Yes	33	2.6
Household income, INR		
< 4000	663	51.3
4001-10,000	536	41.5
> 10,000	93	7.2
Gravida		
Primigravida	695	53.8
Multigravida	597	46.2
Infant gender		
Girl	614	47.5
Boy	671	51.9
Twins	7	0.5
Birthweight		
Normal weight (> 2500 g)	793	61.4
Low birthweight (≤ 2500 g)	499	38.6
Antenatal visits		
< 4	133	10.3
5–6	241	18.7
7–10	900	69.7
> 10	18	1.4
Happy with infant gender		
No	153	11.8
Yes	1139	88.2
Birth attendant		
Traditional Birth Attendant	24	1.9
Relative	25	1.9
Auxiliary Nurse Midwife	129	10.0
Doctor/Nurse	1114	86.2
Delivery place		
Home	51	3.9
Sub-center/PHC/District Health	732	56.7
Maternity/Private Nursing Home	509	39.4
Delivery type		
Vaginal	1074	83.1
Caesarian	218	16.9
Assistance during breastfeeding		
No	435	33.7
Yes	857	66.3

**Table 2:** Mother-infant dyad characteristics by practice of exclusive breastfeeding in rural southern India (*n =* 1292)

Characteristics	Exclusive breastfeeding (*n* = 627)		Non-Exclusive Breastfeeding (*n* = 665)	
	*n*	%	*n*	%
Maternal age, mean(SD)	21.0 ± 2.8	–	20.6 ± 2.7	–
Maternal education				
No education	102	60.7	66	39.3
Primary (1–8 yrs)	223	43.6	288	56.4
More than primary (> 8 yrs)	302	49.3	311	50.7
Religion				
Hindu	621	51.5	659	49.5
Muslim/others	6	50.0	6	50.0
Employed				
No	614	48.8	645	51.2
Yes	13	39.4	20	60.6
Household income, INR				
< 4000	323	48.7	340	51.3
4001-10,000	256	47.8	280	52.2
> 10,000	48	51.6	45	48.4
Gravida				
Primigravida	336	48.4	359	51.7
Multigravida	291	48.7	306	51.3
Infant gender				
Girl	303	49.4	311	50.7
Boy	320	47.7	351	52.3
Twins	4	57.1	3	42.9
Birthweight				
Normal weight (> 2500 g)	370	46.7	423	53.3
Low birthweight (≤ 2500 g)	257	51.5	242	48.5
Antenatal visits				
< 4	55	41.4	78	58.7
5–6	105	43.6	136	56.4
7–10	456	50.7	444	49.3
> 10	11	61.1	7	38.9
Happy with infant gender				
No	69	45.1	84	54.9
Yes	558	49.0	581	51.0
Birth attendant				
Traditional Birth Attendant	11	45.8	13	54.2
Relative	11	44.0	14	56.0
Auxiliary Nurse Midwife	55	42.6	74	57.4
Doctor/Nurse	550	49.4	564	50.6
Delivery place				
Home	23	45.1	28	54.9
Sub-center/PHC/District Health	344	47.0	388	53.0
Maternity/Private Nursing Home	260	51.1	249	48.9
Delivery type				
Vaginal	514	47.9	560	52.1
Caesarian	113	51.8	105	48.2
Assistance during breastfeeding				
No	213	49.0	222	51.0
Yes	414	48.3	443	51.7

Note: Percents are row percents

### Determinants of exclusive breastfeeding

All of the women in the study reported breastfeeding within 72 h of birth and of those, 627 (48.5%) practiced EBF for the first 6 months of the infant’s life. Table 3 describes the bivariate analysis and multivariable logistic regression examining the factors associated with exclusive breastfeeding. Exclusive breastfeeding was significantly associated with three variables: maternal age, maternal education, and number of antenatal visits. Odds of EBF increased slightly with age (aOR 1.04, 95% CI 1.00, 1.09), no education (aOR 1.56, 95% CI 1.10, 2.21), and with 7–10 antenatal visits (aOR 1.57, 95% CI 1.09, 2.27).

**Table 3:** Determinants of exclusive breastfeeding among mothers in rural southern India (*n* = 1292)

	Unadjusted OR (95% CI)	Adjusted OR (95% CI)
Maternal age, y	1.06** (1.02, 1.09)	1.04* (1.00, 1.09)
Maternal education		
No education	1.54* (1.10, 2.16)	1.56* (1.10, 2.21)
Primary (1–8 years yrs)	0.81 (0.64, 1.01)	0.83 (0.66, 1.04)
More than primary (> 8 yrs)	1.00	1.00
Household income, INR		
< 4000	0.93 (0.61, 1.41)	–
4001-10,000	0.85 (0.55, 1.30)	–
> 10,000	1.00	–
Employed		
No	1.00	–
Yes	1.12 (0.79, 1.59)	–
Religion		
Hindu	1.00	–
Muslim/others	1.71(0.62, 4.73)	–
Gravidity		
Primigravida	1.00 (0.81, 1.24)	–
Multigravida	1.00	–
Infant gender		
Boy	1.00	–
Girl	1.11 (0.91, 1.37)	–
Twins	1.41 (0.31, 6.33)	–
Birthweight		
Normal weight (> 2500 g)	1.00	–
Low birthweight (≤ 2500 g)	1.21 (0.97, 1.52)	–
Antenatal visits		
< 4	1.00	1.00
5–6	1.08 (0.72, 1.63)	1.14 (0.74, 1.74)
7–10	1.50* (1.05, 2.14)	1.57* (1.09, 2.27)
> 10	1.88 (0.71, 4.96)	2.06 (1.77, 5.49)
Happy with infant gender		
Yes	1.00	–
No	1.06 (0.78, 1.46)	–
Birth attendant		
Traditional Birth Attendant	0.75 (0.34, 1.67)	–
Relative	0.72 (0.34, 1.53)	–
Auxiliary Nurse Midwife	0.71 (0.94, 1.43)	–
Doctor/nurse	1.00	–
Delivery location		
Home	0.82 (0.47, 1.41)	–
Maternity/Private nursing home	1.16 (0.81, 2.47)	–
Sub-center/PHC/District Health	1.00	–
Delivery type		
Vaginal	1.00	–
Caesarian	1.17 (0.89, 1.55)	–
Assistance during breastfeeding		
Yes	1.00	–
No	1.01 (0.82, 1.26)	–

*p < 0.05, **p < 0.01

The most frequently cited reason for not practicing EBF was “*I thought I would not have enough milk*” (188/665, 28.3%). Other reasons for not breastfeeding included beliefs such as, “*My baby is old enough that he/she no longer needs to be breastfed exclusively*” (8/665, 1.2%) and “*My baby was sick and could not be breastfed*” (3/665, 0.5%). Table 4 describes supplemental feeding among mothers who did not practice exclusive breastfeeding. Mothers supplemented breastmilk with formula or animal milk (42.6%), castor oil or ghee (18.4%), water (24.5%) and/or sugar water or honey (18.8%).

**Table 4:** Supplemental feeding among non-EBF mothers in rural southern India (*n* = 665)

Breastmilk alternative	Yes	No
	*n* (%)	*n* (%)
Formula or animal milk	283 (42.6)	382 (57.4)
Water	163 (24.5)	502 (75.5)
Sugar water or honey	125 (18.8)	540 (81.2)
Castor oil or ghee	122 (18.4)	543 (81.7)

## Discussion

In this study of mother-infant dyads from a rural region of southern India, less than half of mothers practiced exclusive breastfeeding, which is lower than the national rate of 56.0% and Karnataka’s rural population rate of 58.2% [16]. The reasons for low rates of EBF among this population are unknown and warrant further study. The low rate of EBF is particularly concerning given the large percentage of preterm births (43.2%) found in this study and makes increasing exclusive breastfeeding an urgent priority. Similar to findings in other breastfeeding studies conducted in India and globally, EBF rates increased with greater maternal age [17–21]. Our finding that women who delivered via caesarian section had higher odds of EBF though not statistically significant, differ from previous findings. Women with caesarian sections may have higher odds of EBF due to longer time spent in the hospital and perhaps greater opportunity to be coached in breastfeeding practices by healthcare providers. A range of 7 to 10 antenatal visits was associated with the highest odds of exclusive breastfeeding. Studies on the association between EBF and number of antenatal visits in India and other regions of South Asia are discordant [9, 19]. The benefits of breastfeeding should be emphasized during the initial antenatal visit to increase EBF rates among all mothers. Contrary to a breastfeeding study based on a nationally representative sample [19], lower education conferred greater odds of EBF in the present study. In general, higher rates of breastfeeding are associated with lower income and less formal education [9]. For women in rural Karnataka, we suspect higher odds of breastfeeding among women with no education to be related to the economic benefits of breastfeeding.

In the present study, the mothers most frequently reported discontinuation of EBF due to insufficient breastmilk. Mothers most frequently used complementary foods such as infant formula, animal milk, or water to wean or terminate breastfeeding. The feeling that breastmilk was insufficient is a common phenomenon worldwide and a typical response is to give the infant supplements from a very early age [18, 22, 23]. The mother’s concern about milk insufficiency may be explained by poor education regarding techniques to increase breastmilk. Despite these widespread concerns women are encouraged to continue breastfeeding as maternal milk production is finely tuned to the demand of the infant and therefore, consistent and exclusive breastfeeding is critical for stimulating milk production [24].

This study fills an important gap in knowledge regarding prevalence and determinants of EBF among rural women in the southern India. The large sample size and low loss to follow up rate confers greater statistical power and generalizability. The questionnaire was based on validated items from the India’s National Family Health Survey-3 [16]. There are limitations that should be considered when interpreting the results of this study. Participants were asked about breastfeeding practices at 15 days and 6 months after birth. It is possible that there is some degree of recall bias associated with the timing of the interviews and social desirability bias as some respondents may have over reported exclusive breastfeeding. The sample of mothers and infants was drawn from a single subdistrict in southern India, limiting the generalizability of this data to other regions of the world. In addition, the study did not assess physiological, psychological and social factors thought to influence breastfeeding. Future studies should explore factors such as parents and in-laws perceptions of breastfeeding [17], postnatal counseling [17], cultural and traditional practices, and nipple problems among rural South Indian women to identify where potential interventions could be initiated.

## Conclusions

The results of this study reveal the need for health promotion to focus on the benefits of exclusive breastfeeding for both the infant and mother. Education should target new mothers and family members as the previous research suggests a strong influence of mother’s parents and in-laws on child rearing practices [17]. Breastfeeding education provided during pregnancy should be evaluated so that mothers learn the best techniques and benefits of breastfeeding.

## Abbreviations

aOR: Adjusted odds ratio
EBF: Exclusive breastfeeding
LMICs: Low and middle-income countries
PHRII: Public Health Research Institute of India
SD: Standard deviation
WHO: World Health Organization
